# Supplementary material for: Second-Hand Tobacco Smoke Exposure and Smoke-Free Environments in Ethiopia: A Scoping Review and Narrative Synthesis
Source: Int J Environ Res Public Health. 2022 Jul 9;19(14):8404. doi: 10.3390/ijerph19148404 (PMC9324201; doi:10.3390/ijerph19148404)
Supplement: Supplementary file 1 [file ijerph-19-08404-s001.zip › Table S1. Search strategies.pdf]

Table S1: Search strategies

Database	Search terms and steps
**Ovid MEDLINE(R) ALL <1946 to January 18, 2022>**	1) passive smok*.mp. 4908<br>2) exp Environmental Exposure/ or exp Tobacco Smoke Pollution/ or tobacco smoke.mp.343541<br>3) environmental exposure/ and smoke/ 535<br>4) ((secondhand or second-hand or second hand) adj2 smok$).ti,ab. 4462<br>5) (passive adj2 smok$).ti,ab. 5071<br>6) (involuntary adj2 smok$).ti,ab. 134<br>7) (exposure adj2 smok$).ti,ab. 11882<br>8) environmental tobacco smoke.ti,ab.3578<br>9) (exposure adj2 (ETS or SHS)).ti,ab. 2597<br>10) cigarette smoke.mp. 13173<br>11) or/1-10 359374<br>12) (smoke free or smokefree or smoke-free).ti,ab. 4012<br>13) (smok$ ban$ or smok$ rule$ or smok$ restrict$).ti,ab. 1933<br>14) exp Smoke-Free Policy/ 1122<br>15) ((smoke free or smokefree or smoke-free) adj2 (environment$ or place$ or premises or space$)).ti,ab. 617<br>16) ((smoke free or smokefree or smoke-free) adj2 (bar or bars or club or clubs or home$ or office$ or restaurant$ or workplace$)).ti,ab. 750<br>17) ((smoke free or smokefree or smoke-free) adj2 (automobile$ or car$ or vehicle$)).ti,ab. 88<br>18) ((smoke free or smokefree or smoke-free) adj2 (clinic$ or hospital$ or ward$)).ti,ab. 181<br>19) ((smoke free or smokefree or smoke-free) adj2 medical centre$).ti,ab. 0<br>20) ((smoke free or smokefree or smoke-free) adj2 health facility$).ti,ab. 0

	21) ((smoke free or smokefree or smoke-free) adj2 medical center$).ti,ab. 3<br>22) or/12-21 5621<br>23) 11 or 22 362088<br>24) Ethiopia.mp. or exp Ethiopia/ 23327<br>25) 23 and 24 346
**Embase <1974 to 2022 January 18**	1) Tobacco smoke pollution.mp. or exp passive smoking/ 14290<br>2) cigar smoking/ or exp cigarette smoke/ 14331<br>3) tobacco smoke.mp. or exp tobacco smoke/ 21110<br>4) environmental exposure/ and smoke/ 810<br>5) ((secondhand or second-hand or second hand) adj2 smok$).ti,ab. 5675<br>6) passive adj2 smok$).ti,ab. 6844<br>7) (involuntary adj2 smok$).ti,ab. 160<br>8) (exposure adj2 smok$).ti,ab. 16424<br>9) (exposure adj2 (ETS or SHS)).ti,ab. 3183<br>10) or/1-9 53132<br>11) (smoke free or smokefree or smoke-free).ti,ab. 4760<br>12) (smok$ ban$ or smok$ rule$ or smok$ restrict$).ti,ab. 2257<br>13) ((smoke free or smokefree or smoke-free) adj2 (environment$ or place$ or premises or space$)).ti,ab. 765<br>14) ((smoke free or smokefree or smoke-free) adj2 (bar or bars or club or clubs or home$ or office$ or restaurant$ or workplace$)).ti,ab. 834<br>15) ((smoke free or smokefree or smoke-free) adj2 (automobile$ or car$ or vehicle$)).ti,ab. 101<br>16) ((smoke free or smokefree or smoke-free) adj2 (clinic$ or hospital$ or ward$)).ti,ab. 229<br>17) ((smoke free or smokefree or smoke-free) adj2 medical centre$).ti,ab. 0<br>18) ((smoke free or smokefree or smoke-free) adj2 medical center$).ti,ab. 3

	19) or/11-18 6338<br>20) 10 or 19 56604<br>21) exp ethiopia/ or exp ethiopian/ 21801<br>22) ethiopia.mp. 24153<br>23) 21 or 22 24445<br>24) 20 and 23 34
**APA PsycInfo <1806 to January Week 2 2022>**	1) exp Passive Smoking/ or tobacco smoke pollution.mp. 1838<br>2) tobacco smoke.mp. 2251<br>3) ((secondhand or second-hand or second hand) adj2 smok$).ti,ab. 1240<br>4) (passive adj2 smok$).ti,ab. 381<br>5) (involuntary adj2 smok$).ti,ab. 18<br>6) (exposure adj2 smok$).ti,ab. 1885<br>7) environmental tobacco smoke.ti,ab. 412<br>8) (exposure adj2 (ETS or SHS)).ti,ab. 559<br>9) or/1-8 3923<br>10) (smoke free or smokefree or smoke-free).ti,ab. 1489<br>11) (smok$ ban$ or smok$ rule$ or smok$ restrict$).ti,ab. 828<br>12) ((smoke free or smokefree or smoke-free) adj2 (environment$ or place$ or premises or space$)).ti,ab. 187<br>13) ((smoke free or smokefree or smoke-free) adj2 (bar or bars or club or clubs or home$ or office$ or restaurant$ or workplace$)).ti,ab. 263<br>14) ((smoke free or smokefree or smoke-free) adj2 (automobile$ or car$ or vehicle$)).ti,ab. 28<br>15) ((smoke free or smokefree or smoke-free) adj2 (clinic$ or hospital$ or ward$)).ti,ab. 56<br>16) ((smoke free or smokefree or smoke-free) adj2 medical centre$).ti,ab. 0<br>17) ((smoke free or smokefree or smoke-free) adj2 medical center$).ti,ab. 0

	18) or/10-17 2093<br>19) 9 or 18 5125<br>20) Ethiopia.mp. 2075<br>21) 19 and 20 4
