# Supplementary material for: Second-Hand Tobacco Smoke Exposure and Smoke-Free Environments in Ethiopia: A Scoping Review and Narrative Synthesis
Source: Int J Environ Res Public Health. 2022 Jul 9;19(14):8404. doi: 10.3390/ijerph19148404 (PMC9324201; doi:10.3390/ijerph19148404)
Supplement: Supplementary file 1 [file ijerph-19-08404-s001.zip › Table S2.List of invited stakeholder organisations.pdf]

Table S2: List of invited stakeholder organisations

Organization	Category
Addis Ababa University (AAU), Department of preventive medicine	Academia-Government
AAU, Department of Psychiatry	Academia-Government
Campaign for Tobacco Free Kids	Civil Society Organisation (CSO)
Ethiopia Food and Drug Administration (EFDA)	Program-Government
Ethiopia Public Health Institute (EPHI)- Nutrition and Food Science Research Directorate	Research-Government
EPHI-National Data Management Centre	Research-Government
EPHI-Health System Research Directorate	Research-Government
Hawassa University, College of Medicine and Health Sciences	Academia-Government
Health Development and Anti Malaria Association	CSO
Jimma University, College of Health Sciences & Medicine	Academia-Government
Mathewos Wondu Ethiopia Cancer Society	CSO
MeQuamia Community Development Organization	CSO
Ministry of Health	Program-Government
University of Gondar, School of Pharmacy	Academia-Government
World Health Organization (WHO)	UN Organisation
